# Implementation of web-based respondent driven sampling in epidemiological studies

**DOI:** 10.1186/s12874-023-02042-z

**Published:** 2023-10-02

**Authors:** Pedro Ferrer-Rosende, María Feijoo-Cid, María Isabel Fernández-Cano, Sergio Salas-Nicás, Valeria Stuardo-Ávila, Albert Navarro-Giné

**Affiliations:** 1 Research group on psychosocial risks, organization of work and health, Universitat Autònoma de Barcelona, Cerdanyola del Vallès, Spain; 2 Unitat de Bioestadística, Facultat de Medicina, Departament de Pediatria, d’Obstetrícia i Ginecologia i de Medicina Preventiva, Universitat Autònoma de Barcelona, Avda. Can Domènech S/N, Cerdanyola del Vallès, 08193 Spain; 3 Programa de doctorat en Metodologia de la Recerca Biomèdica i Salut Pública, Universitat Autònoma de Barcelona, Cerdanyola del Vallès, Spain; 4 Nursing Department, Faculty of Medicine, Universitat Autònoma de Barcelona, Cerdanyola del Vallès, Spain; 5 Multidisciplinary Research Group in Health and Society (GREMSAS), Barcelona, Spain; 6 Instituto de Salud Pública, Universidad Andrés Bello, Santiago, Chile; 7 Institut d’Estudis del Treball, Universitat Autònoma de Barcelona, Cerdanyola del Vallès, Spain

**Keywords:** Epidemiologic methods, Hard-to-reach populations, Web based sampling, Respondent-driven sampling, WebRDS

## Abstract

**Background:**

Respondent-driven sampling (RDS) is a peer chain-recruitment method for populations without a sampling frame or that are hard-to-reach. Although RDS is usually done face-to-face, the online version (WebRDS) has drawn a lot of attention as it has many potential benefits, despite this, to date there is no clear framework for its implementation. This article aims to provide guidance for researchers who want to recruit through a WebRDS.

**Methods:**

Description of the development phase: guidance is provided addressing aspects related to the formative research, the design of the questionnaire, the implementation of the coupon system using a free software and the diffusion plan, using as an example a web-based cross-sectional study conducted in Spain between April and June 2022 describing the working conditions and health status of homecare workers for dependent people.

**Results:**

The application of the survey: we discuss about the monitoring strategies throughout the recruitment process and potential problems along with proposed solutions.

**Conclusions:**

Under certain conditions, it is possible to obtain a sample with recruitment performance similar to that of other RDS without the need for monetary incentives and using a free access software, considerably reducing costs and allowing its use to be extended to other research groups.

**Supplementary Information:**

The online version contains supplementary material available at 10.1186/s12874-023-02042-z.

## Background

Respondent-driven sampling (RDS) is a sampling method that has gained popularity in epidemiological studies over the years for hard-to-reach populations or those without a sampling frame [1, 2]. This method is based on a chain-referral process, which involves three main steps: formative research, data collection, and data analysis [3].

Formative research is a crucial phase where researchers delve into social network properties of the target population, evaluate the acceptability of RDS as a viable sampling method, determine the selection of initial members (or ‘seeds’), and address survey logistics, including incentives and coupon design [4].

During the data collection process, these ‘seeds’ are required to answer the survey, which should include one or more questions about the size or ‘degree’ of their personal network (e.g., *‘How many people with the characteristics of the target population of the study do you know and/or can you contact right now?‘*). This information is crucial, as RDS estimates use it to calculate the probability of selection for each respondent. Additionally, it is recommended to include some other questions that can serve as diagnostics for the sampling method at the end of the study (for a comprehensive discussion on diagnostics, see Gile et al. 2015) [5].

Once the survey is completed, ‘seeds’ are instructed to recruit a limited number of participants from their personal network, usually through a coupon system. These recruits are asked to take the survey and subsequently become recruiters themselves. This process continues for as many waves as necessary until the desired sample size is reached, the participants’ characteristics have stabilized, or until the recruitment chains become extinct. For the effectiveness of the sampling method, it is ideal for the seeds to have a large and diverse social network.

For data analysis, there is a growing theoretical framework with several proposed estimators for RDS data which under certain assumptions can generate asymptotically unbiased population estimates [6]. Consequently, it becomes vital for researchers to carefully select the RDS inference approach that provides more robust estimates, taking into account the assumptions met by study design [7].

Although RDS has traditionally been conducted face-to-face, the online version (WebRDS) has gained significant attention in the last decade due to its potential benefits over face-to-face RDS [8, 9]. WebRDS offers easy access for participants and ensures anonymity, eliminating time and location-related barriers. Moreover, it allows data collection within a short time frame and at low cost, providing a more efficient recruitment medium [10]. However, similar to other web survey methods, WebRDS may also encounter challenges, such as bias from differential internet access, the possibility of multiple responses, concerns regarding the credibility of online research, and the absence of face-to-face interactions [11].

Despite the increasing use of WebRDS, to our knowledge there is no clear framework for the implementation of this online recruitment method, which is of great importance considering that online approach could be almost as feasible and effective as face-to-face RDS if the population and the application of the method is appropiate [9]. This article aims to provide guidance for researchers who want to recruit through a WebRDS. It covers various aspects, including formative research, implementation of the coupon system using a free acces software, monitoring strategies throughout the recruitment process, and potential problems, along with proposed solutions.

## Development

### Example

We demonstrate the use of WebRDS in the formative research, implementation, and follow-up recruitment phases through data from the CUIDÉMONOS Project [12], a web-based cross-sectional study conducted in Spain from April to June 2022. The objective of the project was to describe the working conditions and health status of homecare workers for dependent individuals. Homecare workers provide routine personal assistance in daily activities to people who require it in private homes. Research indicates that exposure to harmful working conditions can adversely affect the health of workers in the homecare sector [13]. However, despite the growing social and economic significance of this sector, scientific evidence on their working conditions and its impact on occupational health remains scarce. The fact that homecare workers develop their job within private homes further complicates the assessment of their workplace exposures, as there is no sampling frame available.

Given the absence of a sampling frame, we proposed WebRDS as a suitable sampling method for this population. This decision was reinforced by the apparent high motivation of the target population towards the study, the opportunity to collect data from a large geographical area (all of Spain), and the fact that formative research indicated this population is well-connected and regularly uses mobile phones.

### Formative research

As part of our preparatory work, we conducted a comprehensive literature review of previous research and held interviews and focus groups with the invaluable support of a national homecare workers association. These meetings served two primary purposes: first, to evaluate the characteristics of the social network within the target population and assess its suitability for the proposed sampling method, and second, to identify suitable ‘seeds’ for the study. Additionally, these interactions proved instrumental in refining the final questionnaire to encompass the objectives of the study while aligning with the interests and concerns of homecare workers. This alignment played a crucial role in motivating their active participation.

For seed selection, we specifically targeted workers from diverse profiles based on age, gender, migratory status, size of geographic working area, and type of contract, ideally, with a large social network. Considering that long chains of a few seeds are preferable over short chains of many seeds [3, 14] a total of eight seeds were recruited at the beginning and three coupons were allowed per recruiter.

After selecting the seeds, we conducted virtual meetings with them, providing detailed explanations of the study and the recruitment method. Furthermore, we created promotional videos for the study, which were disseminated on YouTube, Facebook groups of homecare workers, and shared through WhatsApp.

### Designing the questionnaire

The questionnaire and coupon system were implemented using the free-to-use application Limesurvey (https://www.limesurvey.org). The questionnaire was based on previously validated instruments in Spain and tailored to align with the specific study objectives. It encompassed a range of essential aspects, including sociodemographic information, labor management practices, psychosocial exposure at work, and health status. Furthermore, following the recommendations of Gile (2015) [5], we included specific questions to capture participants’ self-reported network size:

1. *How many homecare workers do you know in Spain?*
2. *How many of those “n” people could you invite to participate in the survey right now because you have their phone or email contact?*

Response to the second question was the degree used for estimations. Also, to account for finite population and reciprocity assumptions respectively we asked the following questions:

3) *Besides the person who gave you the link, how many other homecare workers do you know who have already participated in this study?*
4) *Would the person who sent you the link to participate in the survey be one of your three contacts if you had received it from someone else?*

Ensuring clear instructions for new recruits was of utmost importance, as it would not be possible to deliver them personally. To address this, we included a brief description of the study along with a five-minute video on the first screen of the survey, before obtaining consent from participants. This video provided detailed explanations of the research method and the recruitment process (https://www.youtube.com/watch?v=tN9M4abXczM&t). Additionally, we provided the contact information of the research team, including an email address and phone number, in case participants had any questions or doubts (see Figure S1).

### Coupon system

**Fig. 1:**
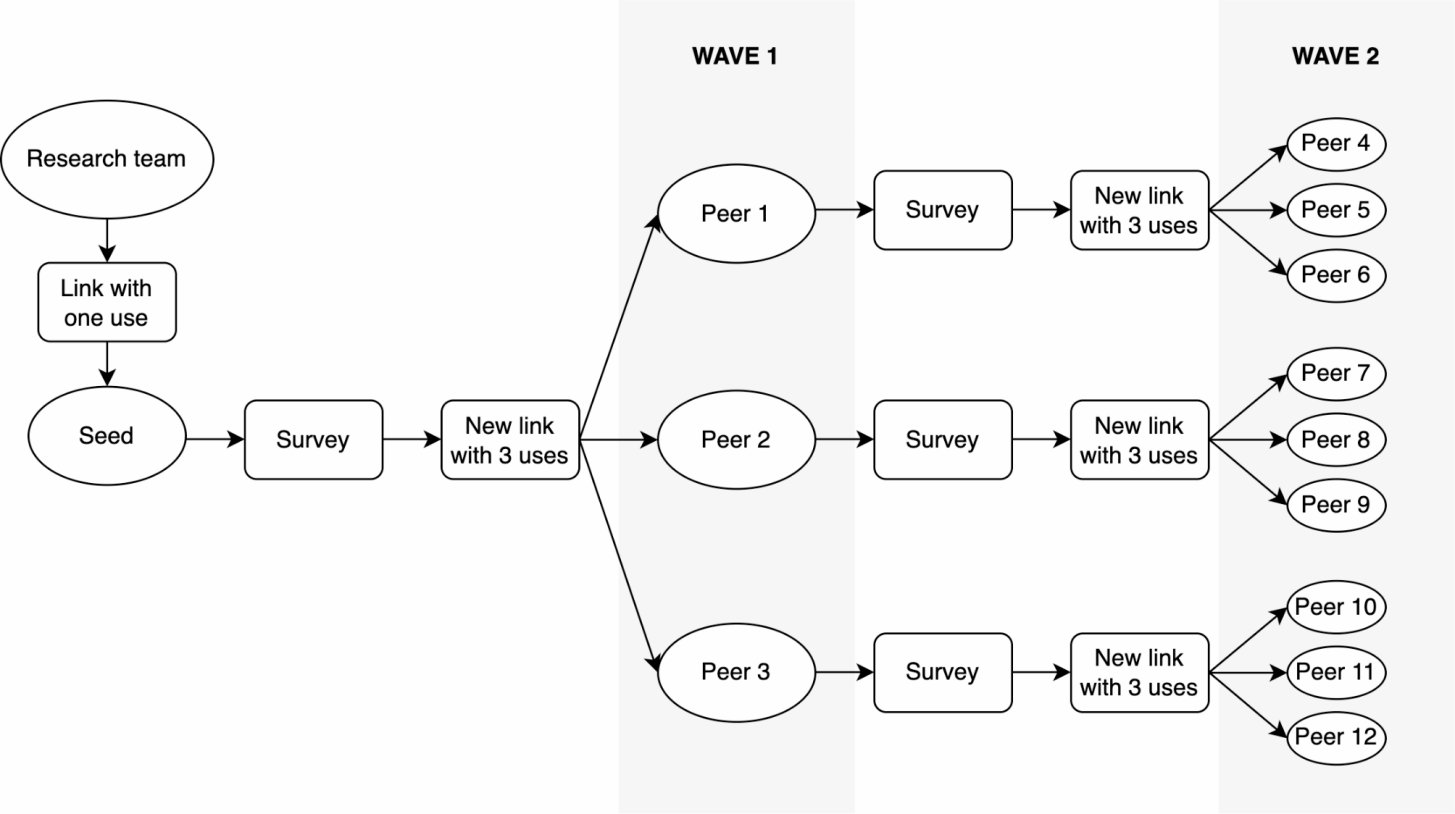
Recruitment process using Web based Respondent-Driven Sampling (WebRDS) from a ‘seed’ respondent

One of the most challenging and crucial aspects of designing the study was establishing the coupon distribution system, which plays a vital role in the success of the method. To accomplish this, a unique and personalized link was automatically generated for each participant after they completed the survey, with a maximum of three uses. Once a participant received and completed the survey through their link, a new link was automatically generated and provided to them, which they could then share with three other individuals, and so on (Fig. 1).

**Fig. 2:**
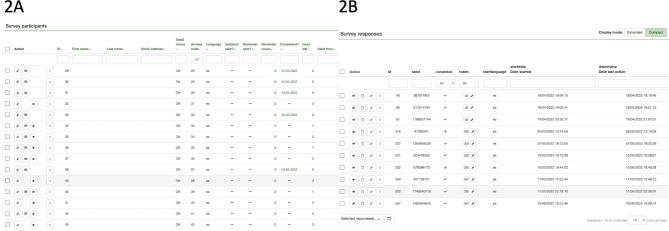
Coupon system in Limesurvey, **2A**: Survey participant list **2B**: Survey responses. Each access code in 2A is represented by the “token” variable in 2B with a limited amount of uses defined by “Uses left” in 2A which is related to a unique personal link. After completion of the survey, the “Id” variable in 2B become the access code for that participant in 2A and therefore the token variable for his three recruited new participants. In this example, participants with Id = 48, 66 and 67 in 2B were invited by participant Id = 30 which invitation had 3 uses (2A), now each of them has 3 invitations that will be reflected in the survey participant list with access codes 48, 66 and 67 respectively

To facilitate this process, we utilized Limesurvey to create a participants database, where each recruiter’s identification number (Id) was linked to a predefined access code or “token,“ corresponding to a unique link with a maximum of three uses. Additionally, a “token” variable was automatically generated in the response database, enabling us to identify the recruiter’s ID for each participant (Fig. 2).

At the conclusion of the survey, participants were presented with two options to share the generated link (Figure S3). The first option allowed them to simply press a box, which automatically opened WhatsApp on their phone or computer, enabling them to send the link to their contacts along with a pre-defined message encouraging participation in the study. The message also provided an explanation of the study methodology and included contact information in case of any doubts (Figure S4). The second alternative was to manually copy the link and share it with three different individuals. Upon survey completion, participants were given the option to provide their contact information, facilitating resolution of any potential issues or future communication (Figure S2).

### Diffusion plan

The success of the project relied heavily on effective promotion before and during recruitment. Several videos featuring homecare worker platform members were shared on social media platforms. The first video was released before the study began, aiming to motivate participation among homecare workers. A second video followed, explaining the recruitment process. Two additional videos were published after recruitment started, encouraging participation with the slogan “No rompas la cadena” (“Don’t break the chain”), visually illustrating the consequences if a branch of the recruitment chain did not recruit. Homecare workers actively participated in sharing these videos to promote the study.

## Application

### Launching day

On the day scheduled for the start of the study, a virtual meeting was conducted with the seeds to discuss final details and make sure that everyone was ready and informed about the recruitment process. After the meeting, everyone received their respective link to the survey through WhatsApp since this would be the preferred platform for sharing the links generated afterwrds.

### Follow-up

During the recruitment process, constant monitoring and evaluation of the system’s performance were crucial. To achieve this, a semi-automated report was generated every two days using “RDS” package of R software [15]. This report provided a descriptive table of the characteristics of the sample and visual representation of the recruitment chain stratified by variables of interest. Additionally, on a weekly basis, the convergence for specific variables of interest was assessed using convergence and bottleneck plots. These ongoing evaluations ensured the reliability and accuracy of the data collection process.

To assess the presence of multiple answers, a two-step process was implemented. First, if a participant had provided contact information, it was verified to ensure there were no repetitions. Second, the combination of specific responses was checked across different participants to identify any potential duplicates.

Contact with the seeds throughout the recruitment process was crucial. They were regularly updated of the progress of their recruitment tree (anonymized) to encourage participation. The objective was to empower all seeds to have an influence on the completion at least of the first two waves of recruitment.

Lastly, for participants whose links had available uses after 5 days of completing the survey and had provided their contact information, a message was sent to them. The message informed them of the number of respondents and emphasized the significance of continuing the recruitment chain. Their respective link was also attached to facilitate forwarding if needed.

**Fig. 3:**
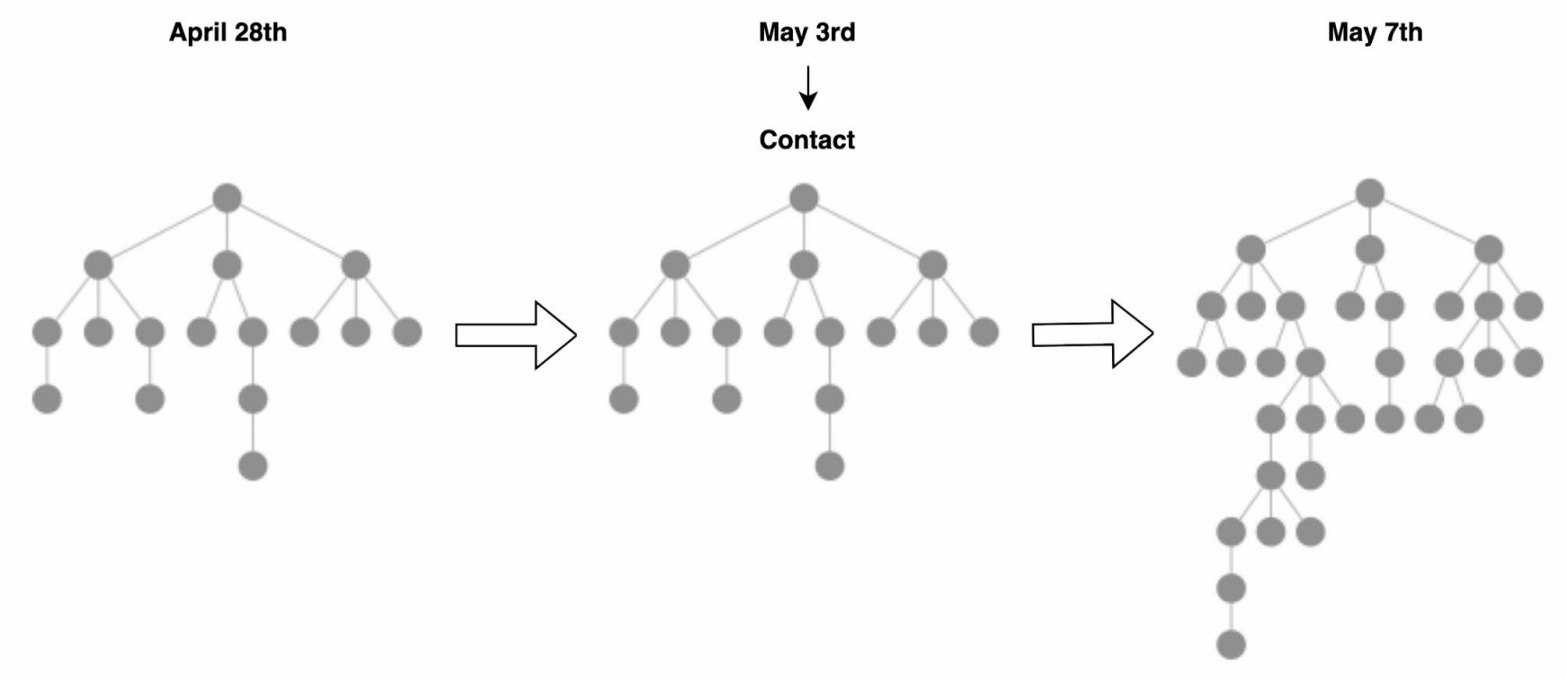
The effect that a single contact had on a recruitment tree of a seed that had not advanced for days

These last steps turned out to be very helpful. An example of this is displayed in Fig. 3, where a recruitment tree of a seed who had not advanced for days is shown and the effect that a single contact had on its growth a few days later.

### Challenges encountered during recruitment process

Despite our efforts to anticipate and address potential challenges during the recruitment process, we encountered a few problems along the way, ranging from technical issues to other diverse natures. Table 1 provides a summary of the main challenges we faced and the solutions we applied to overcome them. Throughout the study, we diligently recorded all these issues in a study log, and if necessary, we made corrections in the analysis. It is worth noting that the high number of participants who left their contact information proved to be invaluable in resolving most of these problems, as we were able to communicate directly with them and find suitable resolutions.

### Recruitment performance

**Fig. 4:**
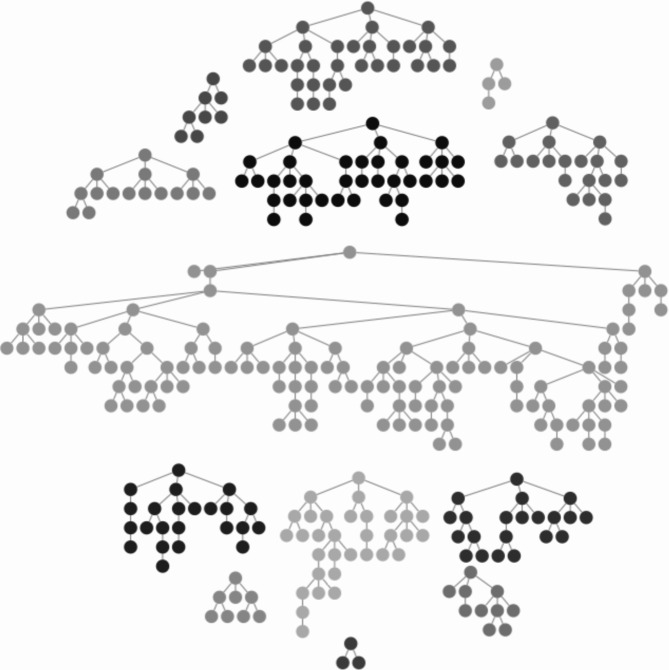
Final recruitment network of the study. (n = 337)

**Fig. 5:**
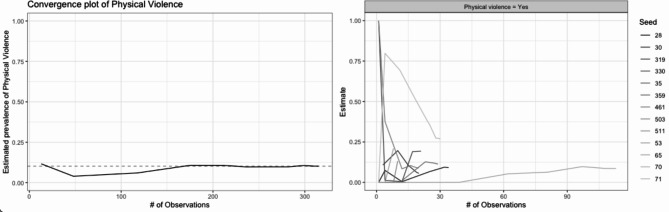
Example of a Convergence and a Bottleneck diagnosis plot

The study initially involved 8 seeds; however, during the recruitment process, we became aware that our focus was primarily on the profiles of the seeds, neglecting their network size and willingness to stay engaged with the research. Consequently, certain initial seeds responded to the survey but failed to maintain contact, leading to unproductive chains. Therefore, we made the decision to introduce new seeds during recruitment that exhibited improved performance (a total of six additional seeds were included). After incorporating those new seeds and removing the only one who did not recruit, we ended up with 337 responses from 13 seeds. Median recruitment chain length (waves) was 4 (Range, 1–10), 162 participants (48.1%) recruited at least one person, 103 (30.6%) recruited at least two and 59 (17.5%) recruited three. The largest recruitment chain contained 123 participants, 36.5% of all recruits. The final recruitment tree and a example of a convergence and a bottleneck diagnosis plots can be seen in Figs. 4 and 5 respectively. Table 2 includes RDS estimates for selected sociodemographic characteristics and recruitment homophily (the ratio of the number of recruits who have the same characteristic as their recruiter to the number we would expect if there was no homophily).

**Table 1:** Issues encountered during recruitment process and the solutions applied

Issue	Description	Solution
Invite link has already been used.	Some participants shared the link that belonged to them, instead of using the one generated specifically after completing the survey.	When this happened, it was addressed by getting the ID of the participant who sent the repeated link. After finding it, the misused token is given one more use. In addition, the participant who sent the wrong link is given his correct link to be forwarded to his contacts. To prevent this from happening, the survey opening message was edited to add clarity to this instruction.
Multiple response	One participant answered the survey three times in a row using her unique link and then using the link she generated because of confusion about the coupon system.	The participant was contacted since she left her contact information, the situation was corrected considering the first response as the official response. Two more uses to the link were given.
	One participant answered the survey in two different time periods through the invitation of two different people.	The participant was contacted since she left her contact information, her first response was considered as the official one and she was asked to tell her recruiter to send another invitation to replace her. One extra use was given to the link.
Distrust for the study	A few participants called us or told recruiters that they have some distrust for the interest of the study.	If the participant contacted us, we tried to assure them of the seriousness of the study, if it was a recruiter who contacted us, we gave them the information to transfer. If the person recruited still did not want to participate, they were asked to invite another person to participate.
Seeds whose recruitment did not advance at the beginning	Some seed’s recruitment trees did not advance beyond the first waves.	Since we had contact with the seeds, we communicated with them by showing them their anonymized recruitment tree so that they could try to secure the first two waves with complete answers. If after a while they did not advance, we added a new seed of same characteristics.
Trouble using the phone	One participant called saying that she did not want to participate because she did not know how to answer the survey on the phone.	We tried to help her through the process but as she showed no interest, she was asked to tell her recruiter to replace her with someone else.

**Table 2:** RDS Estimates and recruitment homophily for some selected sociodemographic characteristics (seeds were removed from the analysis)

Characteristic (N = 324)		N (%)	RDS-II Estimate^a^	Recruitment homophily
Gender				1.03
	Men	18 (5.6%)	4.8 [1.0-13.3]	
	Women	304 (94.4%)	95.2 [86.7–99.0]	
Age				1.12
	39y or less	49 (15.2%)	21.5 [7.0-36.1]	
	40-54y	174 (54.0%)	55.2 [46.0-64.4]	
	55y or more	99 (30.8%)	23.3 [10.4–36.2]	
Place of birth				1.22
	Spain	250 (77.9%)	67.1 [55.3–78.8]	
	Other	71 (22.1%)	32.9 [21.2–44.7]	
Live with partner				1.05
	No	104 (35.9%)	41.3 [26.9–55.7]	
	Yes	186 (64.1%)	58.7 [44.3–73.1]	
Working area size				1.42
	Rural	80 (24.7%)	26.7 [11.3–42.1]	
	Intermediate	99 (30.6%)	34.0 [21.7–46.3]	
	Urban	145 (44.8%)	39.2 [25.3–53.2]	

^a^Bootstrap 95%CI

## Discussion

Conducting RDS sampling online allowed us to obtain a nationwide sample with a total of 338 responses (324 participant after seeds removal). This number of participants is comparable to those reported in face-to-face RDS both in sample size and in number of waves [16]. It was also consistent with that reported in other WebRDS [8]. When comparing sample and RDS-adjusted proportions, we estimate that, without adjusting for RDS, we would be underestimating the prevalence of young individuals and foreigners (Table 2). The recruitment homophily for sociodemographic variables was close to one, indicating that participants tended to recruit others with similar characteristics. However, the homophily was slightly higher in terms of working area size, suggesting that participants were more likely to recruit individuals working in the same area size as themselves.

We attribute the success of this sampling method in our study to four key factors. Firstly, extensive formative research provided us with a profound understanding of the target population and their concerns. This enabled us to design an instrument that not only aligned with the study’s objectives but also intrigued participants to engage without the need for monetary incentives. Secondly, an efficient social media diffusion plan, aided by the active involvement of homecare workers themselves, played a significant role in attracting participants before and during the recruitment process. Thirdly, maintaining consistent communication with the seeds proved crucial in encouraging participation, especially during periods of reduced recruitment and in promptly addressing any issues that arose during the process. Lastly, offering participants the option to provide their contact information allowed us to send reminders and address problems that may have emerged deeper in the recruitment chain. This aspect was particularly vital, given that 54% of the participants chose to share their contact details.

When conducting an RDS participants are usually rewarded for answering the survey (primary reward) and for each person recruited (secondary reward). In our case, we did not provide any rewards, which initially raised concerns about potential participation rates. Fortunately, this was not the case due to the extensive formative research, a population very interested in the study and Permanent contact with the seeds. An advantage of not offering rewards is that it reduces the likelihood of fraud or multiple participation, a concern that arises when conducting WebRDS or any type of online survey [3].

Throughout the recruitment process, we also encountered some challenges. For instance, when selecting seeds, despite acknowledging the significance of network size and diversity, we faced difficulties in reaching suitable seeds that could ensure effective recruitment, particularly for certain profiles. As a result, while not disregarding the desired profiles, we placed greater emphasis on choosing new seeds with large and diverse networks rather than solely focusing on the characteristics of the seed itself [4].

Before implementing the instrument, it is crucial to anticipate any potential issues that may arise during the process and take proactive measures to minimize problems. For instance, we encountered a situation where some participants shared their invitation link instead of the one specifically generated for them to share after completing the survey (Table 1). Fortunately, the software utilized enabled us to make adjustments during the recruitment process, allowing us to provide clearer instructions and consequently reducing the number of participants facing this problem.

One issue that still needs to be addressed in WebRDS is data analysis. There is extensive debate concerning the various mean or variance estimators for RDS [7], as well as regression models used to assess risk factors [17]. These discussions primarily revolve around the potential biases that different methods may exhibit depending on the violation of various RDS assumptions. When applying WebRDS, certain assumptions may be more susceptible to violation compared to face-to-face methods, such as the misspecification of network size. Consequently, it becomes crucial to evaluate which estimator or regression method would perform best, taking into consideration the specific challenges that may arise with this type of RDS sampling.

## Conclusion

In response to the issues of high cost or effort that face-to-face RDS requires, online RDS have emerged as a cost-effective alternative. We propose that under certain conditions, i.e., extensive formative research, good diffusion plan, a population interested in the study and contact with the seeds, it is possible to obtain a sample with recruitment performance similar to that of other RDS without the need for monetary incentives and using a free access software, considerably reducing costs and allowing its use to be extended to other research groups without the need for a large budget.

### Electronic supplementary material

Below is the link to the electronic supplementary material.

Supplementary Material 1

## Data Availability

The datasets used and/or analysed during the current study are available from the corresponding author on reasonable request.
