# Supplementary material for: Implementation of web-based respondent driven sampling in epidemiological studies
Source: BMC Med Res Methodol. 2023 Oct 2;23:217. doi: 10.1186/s12874-023-02042-z (PMC10546631; doi:10.1186/s12874-023-02042-z)
Supplement: Supplementary file 1 — Supplementary Material 1 [file 12874_2023_2042_MOESM1_ESM.pdf]

Supplementary Material

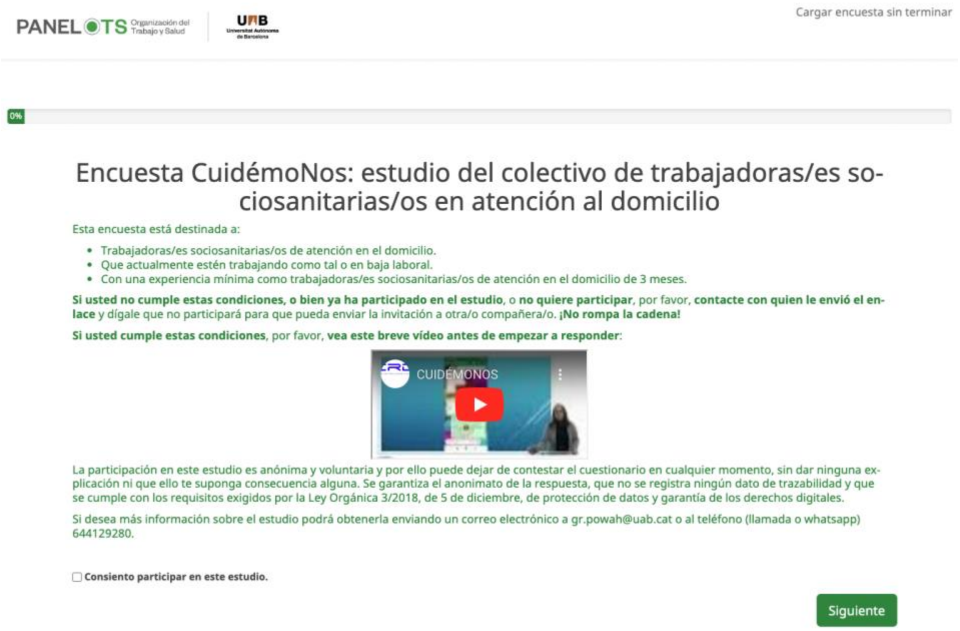
Cargar encuesta sin terminar

0%

Encuesta CuidémoNos: estudio del colectivo de trabajadoras/es sociosanitarias/os en atención al domicilio

Esta encuesta está destinada a:

- Trabajadoras/es sociosanitarias/os de atención en el domicilio.
- Que actualmente estén trabajando como tal o en baja laboral.
- Con una experiencia mínima como trabajadoras/es sociosanitarias/os de atención en el domicilio de 3 meses.

Si usted no cumple estas condiciones, o bien ya ha participado en el estudio, o **no quiere participar**, por favor, **contacte con quien le envió el enlace** y dígale que no participará para que pueda enviar la invitación a otra/o compañera/o. **¡No rompa la cadena!**

Si usted cumple estas condiciones, por favor, **vea este breve vídeo antes de empezar a responder**:

CUIDÉMONOS

La participación en este estudio es anónima y voluntaria y por ello puede dejar de contestar el cuestionario en cualquier momento, sin dar ninguna explicación ni que ello te suponga consecuencia alguna. Se garantiza el anonimato de la respuesta, que no se registra ningún dato de trazabilidad y que se cumple con los requisitos exigidos por la Ley Orgánica 3/2018, de 5 de diciembre, de protección de datos y garantía de los derechos digitales.

Si desea más información sobre el estudio podrá obtenerla enviando un correo electrónico a gr.powah@uab.cat o al teléfono (llamada o whatsapp) 644129280.

☐ **Consiento participar en este estudio.**

Siguiente

Figure S1: First screen of the survey with a brief description of the study and a five-minute video was included in the first screen of the survey prior to consent to participate, explaining in detail the method and the recruitment process (https://www.youtube.com/watch?v=tN9M4abXczM&t). Also, the research team e-mail and phone number were provided in case of any questions or doubts

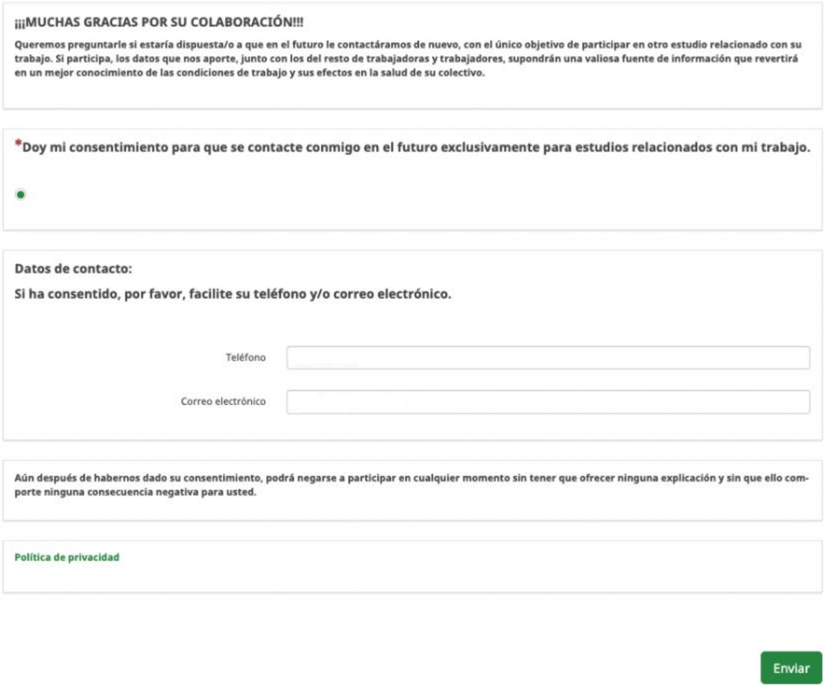
¡¡¡MUCHAS GRACIAS POR SU COLABORACIÓN!!!

Queremos preguntarle si estaría dispuesta/o a que en el futuro le contactáramos de nuevo, con el único objetivo de participar en otro estudio relacionado con su trabajo. Si participa, los datos que nos aporte, junto con los del resto de trabajadoras y trabajadores, supondrán una valiosa fuente de información que revertirá en un mejor conocimiento de las condiciones de trabajo y sus efectos en la salud de su colectivo.

***Doy mi consentimiento para que se contacte conmigo en el futuro exclusivamente para estudios relacionados con mi trabajo.**

Datos de contacto:

Si ha consentido, por favor, facilite su teléfono y/o correo electrónico.

Teléfono

Correo electrónico

Aún después de habernos dado su consentimiento, podrá negarse a participar en cualquier momento sin tener que ofrecer ninguna explicación y sin que ello comporte ninguna consecuencia negativa para usted.

Política de privacidad

Enviar

Figure S2. After completing the survey, the option to provide contact information was given to clarify possible problems or to be reached in the future .

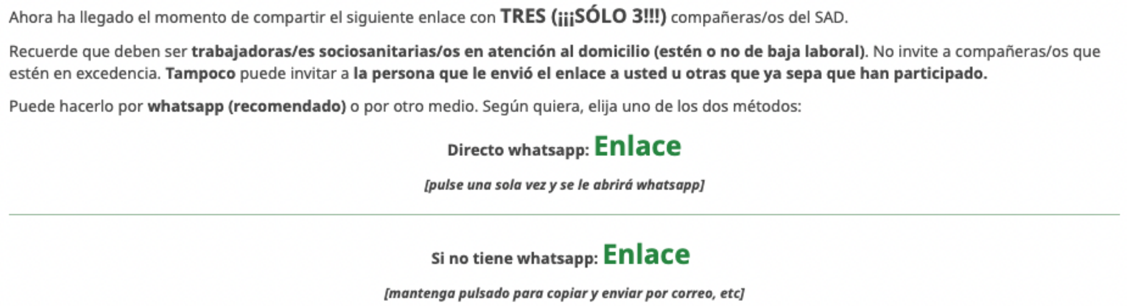

Figure S3. Final message and options to share the generated link at the end of the survey.

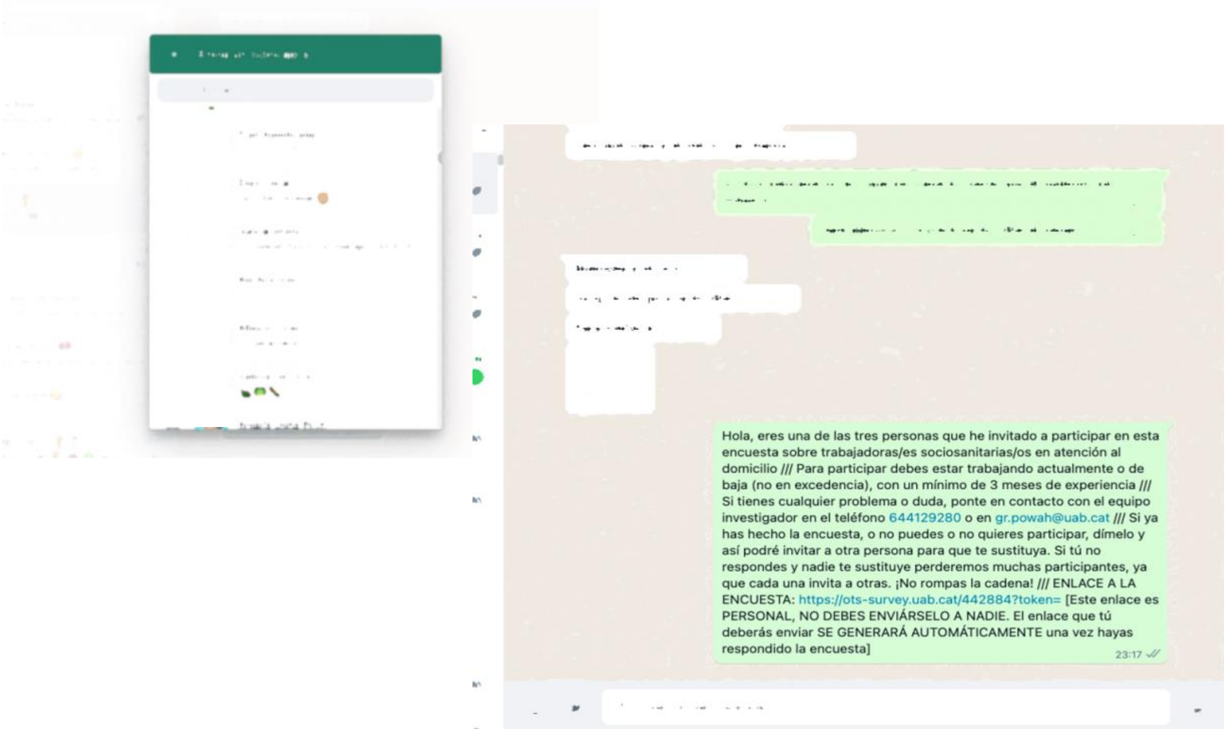

Figure S4. Predefined invitation message sent to each new recruiter encouraging them to participate in the study, explaining the methodology and with contact information in case of any doubts opened automatically for WhatsApp in a computer (This is just an example, not a message sent to a real participant).
